# MRI advances in the imaging diagnosis of tuberculous meningitis: opportunities and innovations

**DOI:** 10.3389/fmicb.2023.1308149

**Published:** 2023-12-11

**Authors:** Xingyu Chen, Fanxuan Chen, Chenglong Liang, Guoqiang He, Hao Chen, Yanchan Wu, Yinda Chen, Jincen Shuai, Yilei Yang, Chenyue Dai, Luhuan Cao, Xian Wang, Enna Cai, Jiamin Wang, Mengjing Wu, Li Zeng, Jiaqian Zhu, Darong Hai, Wangzheng Pan, Shuo Pan, Chengxi Zhang, Shichao Quan, Feifei Su

**Affiliations:** ^1^Department of Infectious Diseases, Wenzhou Central Hospital, Wenzhou, China; ^2^The First School of Medicine, Wenzhou Medical University, Wenzhou, China; ^3^School of Biomedical Engineering, School of Ophthalmology and Optometry, Eye Hospital, Wenzhou Medical University, Wenzhou, China; ^4^Postgraduate Training Base Alliance of Wenzhou Medical University, Wenzhou, China; ^5^Wenzhou Institute, University of Chinese Academy of Sciences, Wenzhou, China; ^6^School of Electrical and Information Engineering, Quzhou University, Quzhou, China; ^7^Baskin Engineering, University of California, Santa Cruz, CA, United States; ^8^Wenzhou Medical University, Wenzhou, China; ^9^Renji College of Wenzhou Medical University, Wenzhou, China; ^10^School of Materials Science and Engineering, Shandong Jianzhu University, Jinan, China; ^11^Department of Big Data in Health Science, The First Affiliated Hospital of Wenzhou Medical University, Wenzhou, China; ^12^Key Laboratory of Intelligent Treatment and Life Support for Critical Diseases of Zhejiang Province, Wenzhou, China; ^13^Zhejiang Engineering Research Center for Hospital Emergency and Process Digitization, Wenzhou, China; ^14^Wenzhou Key Laboratory of Diagnosis and Treatment of Emerging and Recurrent Infectious Diseases, Wenzhou, China; ^15^Department of Infectious Diseases, Wenzhou Sixth People’s Hospital, Wenzhou, China

**Keywords:** tuberculous meningitis, neurological infections, *Mycobacterium tuberculosis*, MRI, artificial intelligence, machine learning

## Abstract

Tuberculous meningitis (TBM) is not only one of the most fatal forms of tuberculosis, but also a major public health concern worldwide, presenting grave clinical challenges due to its nonspecific symptoms and the urgent need for timely intervention. The severity and the rapid progression of TBM underscore the necessity of early and accurate diagnosis to prevent irreversible neurological deficits and reduce mortality rates. Traditional diagnostic methods, reliant primarily on clinical findings and cerebrospinal fluid analysis, often falter in delivering timely and conclusive results. Moreover, such methods struggle to distinguish TBM from other forms of neuroinfections, making it critical to seek advanced diagnostic solutions. Against this backdrop, magnetic resonance imaging (MRI) has emerged as an indispensable modality in diagnostics, owing to its unique advantages. This review provides an overview of the advancements in MRI technology, specifically emphasizing its crucial applications in the early detection and identification of complex pathological changes in TBM. The integration of artificial intelligence (AI) has further enhanced the transformative impact of MRI on TBM diagnostic imaging. When these cutting-edge technologies synergize with deep learning algorithms, they substantially improve diagnostic precision and efficiency. Currently, the field of TBM imaging diagnosis is undergoing a phase of technological amalgamation. The melding of MRI and AI technologies unquestionably signals new opportunities in this specialized area.

## 1 Introduction

Worldwide, tuberculosis (TB) is the 13th leading cause of death and the second leading infectious killer after COVID-19 (WHO, 2023). Tuberculous meningitis (TBM), the most perilous manifestation within the TB spectrum, has seen a rising incidence in recent years, especially among the middle-aged and elderly. Clinically, TBM presents severely, portending poor neurological outcomes, with both disability and mortality rates being considerably high (Török, 2015; Wen et al., 2019; Huang et al., 2022). Children under the age of four, the elderly, and individuals testing positive for HIV stand as the most susceptible populations to TBM infection (Wilkinson et al., 2017; Cano-Portero et al., 2018). Clinically, TBM manifests as a form of subacute meningitis, featuring symptoms like headaches, fever, vomiting, and altered mental status. Intriguingly, these symptoms parallel those of bacterial meningitis and viral meningitis, further complicating the timely diagnostic process. In advanced disease states, the onset of neurological complications sharply exacerbates both mortality and the likelihood of lasting disability linked to TBM (Roca et al., 2008; Török, 2015; Mezochow et al., 2017; Kumar et al., 2021; Xu et al., 2021). Hence, early, precise, and expedient diagnostic and therapeutic interventions are crucial to reducing the mortality associated with this condition.

While lumbar punctures have historically served as the mainstay for TBM diagnosis through clinical evaluation and cerebrospinal fluid analysis, recent advancements in radiological sciences have considerably augmented the diagnostic toolkit. Presently, magnetic resonance imaging (MRI) has emerged as the imaging modality of choice for identifying TBM. Its inherent sensitivity to variations in the water molecule content and molecular environments of different tissues allows MRI to offer unparalleled soft tissue contrast. The brain is composed of a myriad of soft tissues, each with unique water molecule content and structural characteristics. MRI, with its capability to distinctly delineate these tissues, vividly visualizes manifestations such as meningeal inflammation, edema, and tuberculous nodules. As a result, its sensitivity and specificity markedly surpass that of computed tomography (CT) scans. Cutting-edge MRI techniques, such as magnetic resonance angiography and perfusion imaging, have broadened the scope from mere anatomical assessment to include functional and molecular dimensions. This dual functionality serves to heighten diagnostic precision for TBM and facilitates the early detection of complications (Schaller et al., 2019; Baloji and Ghasi, 2020).

The focus of this review is to critically examine MRI technology in the modern context of diagnostic imaging, with an emphasis on its applications and challenges related to TBM. The intent is to furnish radiologists and neurologists with nuanced diagnostic and therapeutic insights.

## 2 Clinical and imaging characteristics of TBM

**Figure 1:**
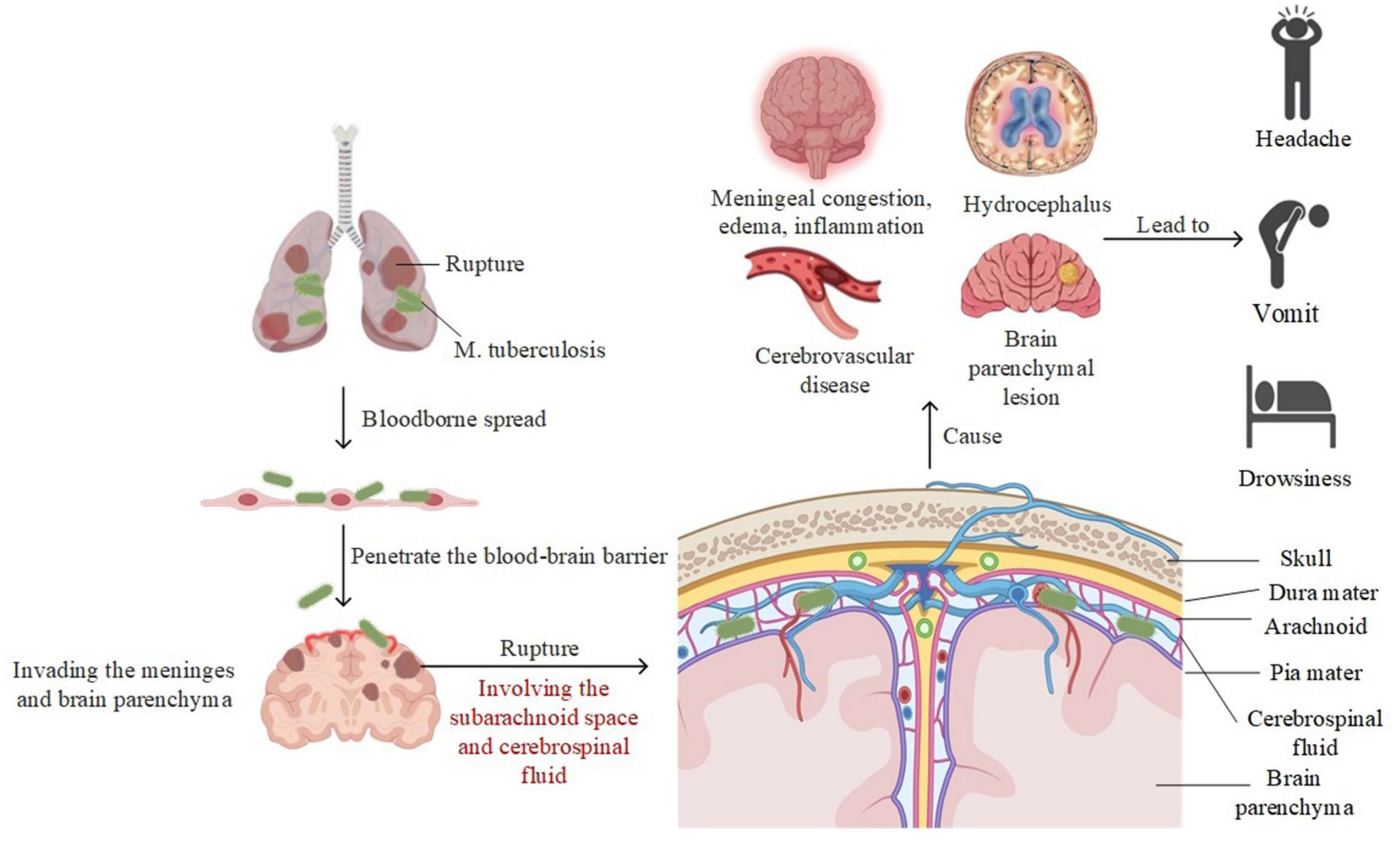
The pathogenesis of TBM.

TBM emanates either from primary or secondary pulmonary tuberculosis infections, catalyzed by the translocation of the *Mycobacterium tuberculosis* complex across the blood–brain barrier, resulting in the formation of nodules in the brain and the invasion of the meninges and brain parenchyma. If these nodules rupture, bacteria can spread further, leading to meningeal congestion, edema, and the release of inflammatory exudates. This can result in conditions such as hydrocephalus, vascular issues, and brain damage, accompanied by clinical symptoms (Figure 1). The prototypical clinical manifestation is subacute meningitis, characterized by a gamut of symptoms that include headache, vomiting, meningeal irritation, focal neurological deficits, visual deterioration, cranial nerve damage, and elevated intracranial pressure. As the disease progresses to advanced stages, the prognosis significantly worsens with the emergence of neurological complications such as epilepsy and stroke (Marais et al., 2010; Solomons et al., 2014). To date, the early diagnostic approaches for TBM still warrant refinement. Peripheral blood leukocytosis, accelerated erythrocyte sedimentation rate, positive tuberculin skin tests, or chest X-rays serve as ancillary indicators for either active or latent tuberculosis infections. Yet, the primary diagnostic fulcrum remains anchored in cerebrospinal fluid analysis and radiological evaluations. Cerebrospinal fluid assays directly detect *Mycobacterium tuberculosis* and pertinent immunological reactions, offering physicians pivotal insights into inflammation and infection through the examination of cellular components, protein concentrations, and glucose levels in the fluid. Nonetheless, inherent challenges such as limited sensitivity and specificity, delayed results, and the inability to directly furnish cerebral structural information render it an imperfect standalone diagnostic tool for TBM (Wilkinson et al., 2017). The advancement of radiological techniques not only serves as a linchpin in the diagnosis but also incisively demarcates complications linked to central nervous system tuberculosis. These radiological methods boast numerous advantages, notably non-invasiveness, speed, and real-time reporting capabilities.

Both CT and MRI have demonstrated irreplaceable utility in evaluating complications linked to TBM. Canonical radiological findings consist of hydrocephalus, basal cistern enhancement, cerebral infarction, diffuse cerebral edema, and tuberculomas. Lu et al. conducted a retrospective analysis of 289 TBM patients and found that 254 (88%) displayed diverse radiological abnormalities. Notably, hydrocephalus was observed in 204 cases, while parenchymal enhancement, contrast enhancement of the basal cisterns, cerebral infarction, and localized or diffuse cerebral edema were also prevalent (Lu et al., 2021). Comparatively, MRI, especially contrast-enhanced MRI scans, showed superior sensitivity and specificity, often revealing meningeal enhancement at earlier stages (Bhasin et al., 2020). Liu demonstrated that, in a research study encompassing 28 confirmed TBM cases, plain MRI scans indicated abnormal meningeal signs in 13 patients, whereas contrast-enhanced MRI scans highlighted significant meningeal enhancement and thickening in 27 patients (Xin, 2019). Distinctive MRI features such as basal ganglia meningeal enhancement, hydrocephalus, cerebral infarction, tuberculomas, and cranial nerve injuries have been seminal in the diagnosis of TBM (Bernaerts et al., 2003).

This corpus of traditional MRI findings has not only been indispensable in the early diagnosis of TBM but also illuminates a more nuanced understanding of the disease’s multifaceted complexities. Amid rapid advancements in medical imaging technologies, our grasp of TBM has now reached novel depths. Emerging MRI techniques enable a more meticulous analysis of both micro and macro features of TBM, thereby heralding a new era in the imaging diagnosis of this disease.

## 3 Advanced MRI technologies in TBM

### 3.1 Vessel wall imaging

Tuberculous cerebral arteritis serves as a frequent complication of TBM, predominantly manifesting as inflammation in small arteries. Tuberculous cerebral arteritis can be instigated by a direct bacterial invasion (Wang et al., 2015), wherein vessels within the circle of Willis become infiltrated by exudates from the basal cisterns, initially affecting the vessel walls, and subsequently propagating to the subendothelial cells, leading to luminal narrowing and thrombosis formation. This condition exacerbates cerebral blood circulation, consequently culminating in cerebral infarction. Conventional vascular imaging modalities, including computed tomography angiography (CTA), magnetic resonance angiography (MRA), and digital subtraction angiography (DSA), excel at lumen visualization but fall short in the nuanced assessment of vessel wall integrity (Pei et al., 2019). Contrastingly, vessel wall imaging (VWI), a sophisticated MRI technique, is specifically designed to investigate the structural and pathological changes occurring within the vessel walls, thereby offering a more holistic view, particularly in evaluating vessel wall enhancement and stenosis.

In a groundbreaking study, Choudhary et al. diagnosed 101 TBM patients. Beyond utilizing standard MRI protocols, they incorporated high-resolution magnetic resonance VWI and time-of-flight magnetic resonance angiography into their imaging suite. Their inquiry extended to second-order arterial branches and performed rigorous statistical analyses related to the staging of TBM, infarctions, and time-of-flight magnetic resonance angiography outcomes. Significantly, as the disease trajectory unfolded, VWI exhibited heightened vessel involvement. The finding that certain arterial enhancements were intimately correlated with infarctions in their respective perfusion territories. In the realm of sensitivity for detecting complications, VWI was demonstrably superior to MRA, although MRA maintained a slight advantage in terms of specificity (Choudhary et al., 2021).

Additionally, a case study led by Feitoza et al. highlighted a confirmed TBM patient. Utilizing high-resolution vessel wall imaging (HR-VWI), they not only supplemented traditional lumen imaging but also unearthed various etiological factors contributing to lumen narrowing. The synergistic employment of MRI and HR-VWI emerged as an instrumental diagnostic apparatus for portraying TBM arteritis, precisely identifying vessel wall enhancements without distorting vessel calibers. Consequently, the incorporation of VWI into mainstream MRI protocols stands as an indispensable asset in the early identification of cerebrovascular complications, thereby undeniably augmenting the prospects for a favorable disease prognosis (Feitoza et al., 2021). However, VWI is not without its limitations. For instance, it often necessitates extended scanning durations, elevating the risk of motion artifacts and potentially exacerbating discomfort for patients in acute phases.

### 3.2 Magnetic resonance venography

Magnetic resonance venography (MRV) constitutes a non-invasive diagnostic modality grounded in the principles of MRI, meticulously designed to visualize and evaluate the venous architecture, particularly that of the cerebral and spinal vasculature. The cornerstone of this technology is its capacity to generate high-resolution images by measuring the differential motion of blood, thereby furnishing healthcare practitioners with robust tools for the diagnosis and management of venous pathologies. Technically, the method is largely anchored in two primary techniques, time-of-flight and phase contrast, with their imaging paradigms predicated upon the signal variances between the coursing blood and the adjacent tissue matrix.

In a comprehensive study led by Bansod et al., cerebrospinal fluid examinations and enhanced MRI assessments were administered to 107 patients, ensuring that each participant underwent time-of-flight MRV. During the subsequent six-month follow-up, a modified Barthel index of ≤12 was employed as a criterion for poor prognosis. Observational data collected 6 months later revealed that 12 patients, constituting 11.2% of the sample, exhibited abnormalities in MRV. The most frequently affected site was identified as the superior sagittal sinus, predominantly influenced by thrombus formation. Intriguingly, through multivariate statistical analyses, symptoms such as vomiting, altered consciousness, seizures, papilledema, visual impairment, and oculomotor nerve palsy were not considered statistically significant. Hence, this suggests that approximately 11% of patients may manifest aberrant signals in MRV, indicative of venous sinus thrombosis, with the superior sagittal sinus being particularly vulnerable (Bansod et al., 2018). Nevertheless, although MRV may have certain advantages in detecting venous sinus thrombosis, it also has some limitations. For example, its reduced sensitivity to small blood vessel and microcirculation changes could result in an inability to detect early minor vascular abnormalities.

### 3.3 Phase-contrast magnetic resonance imaging

Phase-contrast magnetic resonance imaging (PC-MRI) constitutes a specialized modality within the realm of MRI technology, devised to quantify and delineate the velocity and directionality of fluid movement in biological systems, predominantly blood. The crux of this innovative technology lies in the capture of images predicated on tracking phase alterations in protons present within the fluid. These phase changes are directly proportional to the fluid’s velocity and directional flow. The application of PC-MRI is particularly salient in the quantitative measurement of blood flow velocities and directions, rendering it invaluable for cardiovascular and cerebrovascular examinations, as well as the assessment of vascular anomalies like aneurysms, arteriosclerosis, and cerebrovascular accidents.

In an incisive study helmed by Ashta et al., the prowess of PC-MRI was leveraged to evaluate 30 patients diagnosed with TBM, boasting an average age of 24 years, juxtaposed against 30 age and gender-matched healthy volunteers. The investigative focus was chiefly laid on procuring axial scans perpendicular to the aqueduct and mid-sagittal phase-contrast scans designed to quantify fluidic dynamic parameters. These encompassed pivotal metrics such as peak velocity, mean velocity, net forward volume, mean flow, and stroke volume. The results unambiguously elucidated that PC-MRI possesses remarkable sensitivity in furnishing a holistic evaluation of cerebrospinal fluid dynamics in patients afflicted with TBM. The technology provided an amalgam of both qualitative and quantitative data sets. In two particular cases marked by severe hydrocephalus, the cerebrospinal fluid flow exhibited manifest qualitative alterations, most notably the absence of the standard sinus waveforms. On a quantitative front, the TBM patients beset by hydrocephalus manifested discernable numerical disparities in cerebrospinal fluid dynamics in comparison to both their healthy counterparts and TBM patients devoid of hydrocephalus (Ashta et al., 2022). It is worth noting that PC-MRI relies on the correct parameter settings for blood flow measurements, and incorrect settings may lead to inaccurate measurements.

### 3.4 Arterial spin labeling and diffusion tensor imaging

Arterial spin labeling (ASL) epitomizes a functional magnetic resonance imaging (fMRI) modality devised explicitly for the quantification of cerebral blood flow (CBF). Its cardinal merit resides in its non-contrast characteristic, facilitating the tagging of water molecules in blood via magnetic fields alone, with subsequent quantification of these labeled entities within the cerebral milieu. By generating temporal comparisons of blood flow images, researchers are endowed with a profound understanding of the brain’s functional modulations and metabolic conditions. In parallel, diffusion tensor imaging (DTI) manifests as a sophisticated MRI technique tailored for the exploration of the brain’s white matter fiber tract microarchitecture and its interconnectedness. Within the realm of DTI, differential rates and directional tendencies of water molecular diffusion enable the assembly of “diffusion tensor” images, thereby unveiling intricate details about tissue structures. Specifically, mean diffusivity (MD) quantifies the aggregate rate of water molecule diffusion, while fractional anisotropy (FA) captures the unidirectional characteristics of water molecular diffusion, essentially serving as a barometer for the structural integrity of fiber tracts.

In a comprehensive year-long MRI investigation piloted by Kumar et al., a cohort of 30 TBM patients underwent meticulous evaluation utilizing both ASL and DTI modalities. Contrariwise, the indices for MD and FA across the frontal lobe white matter, basal ganglia, thalamus, and pons demonstrated negligible variances when juxtaposed against the control group. Nevertheless, in regions exhibiting infarcts, the FA values manifested a significant decrement among the TBM subjects. Concomitantly, the CBF values within the basal ganglia also displayed a relative diminution among those affected by coincident infarcts. These empirical observations advocate that both ASL and DTI stand as potent tools in identifying deviations in blood flow and white matter fiber integrity among patients diagnosed with TBM (Kumar and Gutch, 2020). However, despite the significant advantages of these techniques, ASL exhibits limited sensitivity to low-perfusion areas, potentially missing certain lesions. TBM can induce lesions in brain white matter that may involve complex fiber crossing, making DTI data interpretation challenging and reducing its value in determining lesion nature and precise localization (Table 1).

**Table 1:** Key advances in imaging techniques for TBM.

Author	Year	Number of patients	Main technology	Clinical applications
Choudhary N et al.	2021	101	MR VWI	VWI into routine MRI protocols significantly enhances early detection and management of cerebrovascular complications, thereby aiding in the improvement of disease prognosis.
Feitoza LM et al.	2021	1	HR-VWI	HR-VWI serves as a potent diagnostic tool for identifying TBM-associated arteritis. It allows for the detection of vessel wall enhancement, even in the absence of changes in vascular diameter.
Bansod A et al.	2018	107	MRV	MRV abnormalities indicating venous sinus thrombosis may occur in approximately 11% of patients. The superior sagittal sinus is most commonly affected. Notably, in patients with TBM, abnormal MRV findings may not necessarily predict an adverse outcome.
Ashta A et al.	2022	30	PC-MRI	PC-MRI exhibits remarkable sensitivity in comprehensively assessing alterations in the cerebrospinal fluid dynamics in patients with Tuberculous TBM, offering both qualitative and quantitative data.
Kumar S et al.	2020	30	ASL and DTI	ASL and DTI are effective in detecting abnormalities in cerebral blood flow and changes in the integrity of white matter fibers in TBM patients.

## 4 The future prospects of artificial intelligence and radiomics in TBM imaging diagnosis

Artificial intelligence (AI), delineated as the orchestration of computational algorithms to emulate human cognitive faculties, diminishes manual intervention to an unparalleled extent. This technological paradigm has been ubiquitously assimilated into various spheres of medical science, encompassing, but certainly not circumscribed to, diagnostic modalities, statistical evaluations, robotic-assisted interventions, and biological modeling. Specifically, machine learning, a specialized sub-discipline of AI, capitalizes on the empirical analysis of sample datasets to architect mathematical models. This signifies that machine learning, as distinguished from conventional computational techniques, affords predictive capabilities and decision-making faculties devoid of the need for overt programming. Deep learning, a nuanced branch of machine learning, focuses intently on the employment of multi-layered neural network architectures to manage intricate data sets and facilitate feature extraction (Currie et al., 2019; Law et al., 2021). In recent years, many kinds of deep learning-based research such as single-cell multi-omics data analysis (Hu et al., 2023; Meng et al., 2023), computational toxicology (Wang et al., 2023), miRNA-lncRNA interaction (Wang et al., 2022), and metabolite-disease associations prediction (Gao et al., 2023) have been carried out in bioinformatics. Emerging as a vanguard technology, AI serves to augment existing diagnostic strategies for TBM. AI possesses the acumen to discern and assimilate complex patterns within voluminous imaging data, patterns that may elude the intuitive grasp of radiologists, thus elevating the precision in detecting TBM-specific imaging hallmarks. Additionally, it mitigates the incidence of diagnostic omissions and errors attributable to human factors, automates the monitoring and tracking of disease progression, and some models are even capable of prognosticating the trajectory of the illness.

In the realm of modern medical science, the pertinence of AI has undergone extensive substantiation, especially within the niche of neuroimaging. A myriad of scholarly endeavors have adeptly employed these technological substrates for multifaceted applications, such as the early diagnosis of neurodegenerative disorders, aiding clinicians in the precise localization and treatment of cerebral neoplasms, and monitoring the prognosis of demyelinating neurological diseases (Ion-Mărgineanu et al., 2017; Kamal et al., 2018; Ranjbarzadeh et al., 2023; Shanmugavadivel et al., 2023). At present, AI has been preliminarily applied to the diagnosis of TBM, for example, by leveraging fuzzy clustering to identify areas of high density associated with TBM in CT scans, utilizing AI algorithms to determine the optimal dosing predictions for levofloxacin within TBM-related treatment protocols, employing machine learning techniques as a discriminating method between TBM and viral meningitis, integrating cerebrospinal fluid metagenomic next-generation sequencing with a host gene expression-based machine learning classifier (Halberstadt and Douglas, 2008; Deshpande et al., 2018; Jeong et al., 2021; Ramachandran et al., 2022). Redirecting the spotlight to the imaging diagnosis of TBM, groundbreaking work steered by Huynh et al. postulates that diagnostic tools predicated on AI and machine learning paradigms are incrementally achieving fruition. These burgeoning tools are designed to equip clinicians with a methodologically rigorous, equitable, and automated framework for cerebral image evaluation, thereby facilitating informed decisions pertaining to therapeutic interventions and prognostic outcomes. It is surmised that machine learning modalities could play an instrumental role in shaping the future landscape of TBM diagnosis and the personalization of treatment regimens (Huynh et al., 2022).

Radiomics, a burgeoning interdisciplinary scientific frontier, is dedicated to the assimilation of medical imaging and molecular biology methodologies to garner a holistic understanding of biological architecture, functional intricacies, and molecular signatures. This interdisciplinary approach has already seen successful applications, such as inferring tumor genotypes and biological pathways from radiomic phenotypes, distinguishing between benign and malignant nodules using radiomic features within and around pulmonary nodules. It is worth noting that in the field of neuroimaging, particularly in TBM, the amalgamation of T2-weighted imaging (T2WI)-based radiomic markers with cutting-edge deep learning algorithms furnishes an automated, non-invasive diagnostic modality proficient in detecting nuanced variations in the basal cisterns in the context of TBM. This avant-garde technique has been adeptly applied to identify subtle morphological changes in the basal cisterns, thus revealing the prodigious diagnostic capabilities of this innovative technology in the realm of TBM (Bi et al., 2019; Nishino, 2019; Ma et al., 2022).

In summary, the amalgamation of AI and radiomics presents a broad spectrum of applications in TBM imaging diagnosis, offering new, effective, and automated tools for future advancements in this field.

## 5 Future directions in TBM imaging diagnosis

**Figure 2:**
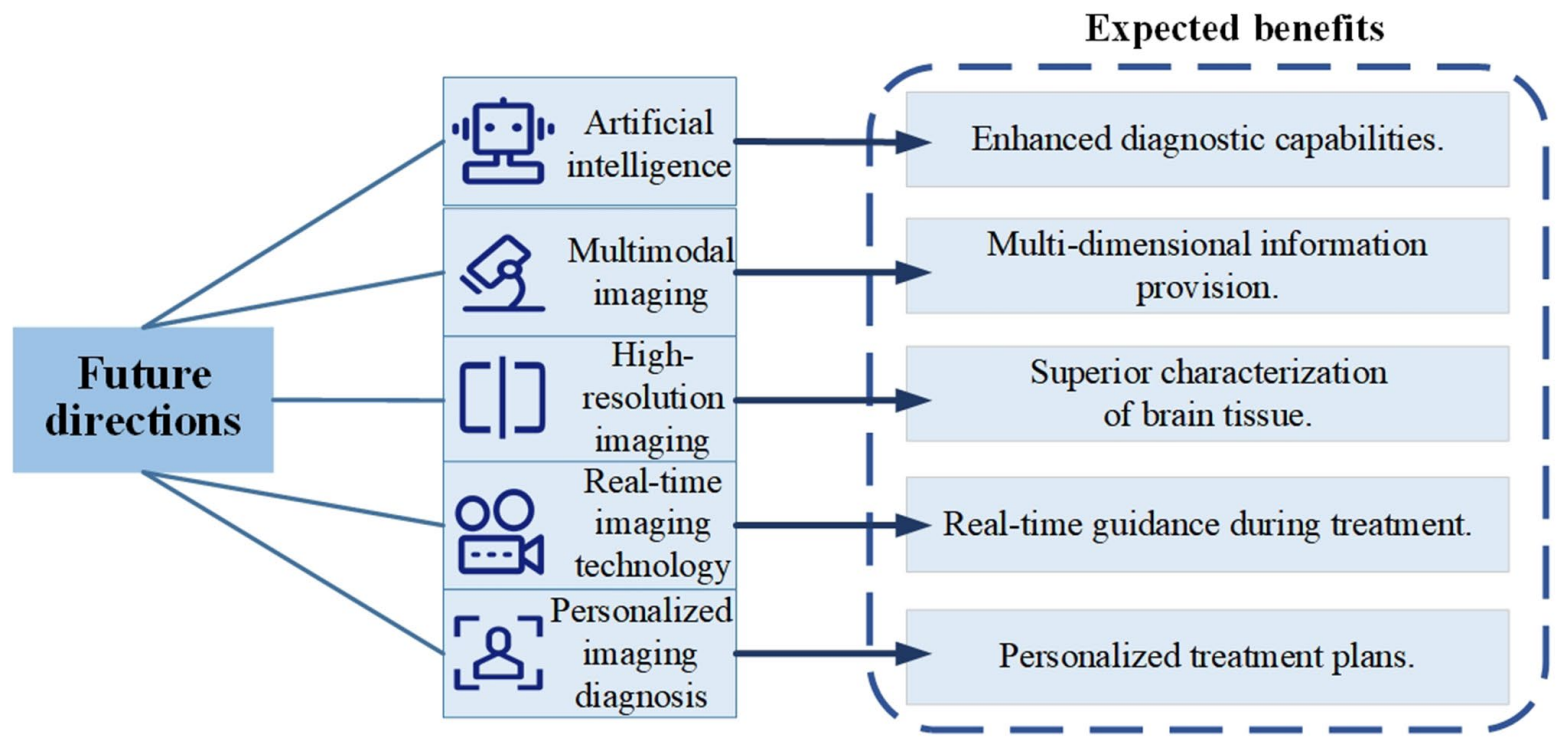
Future directions in TBM imaging diagnosis.

Advances in technology are rapidly reshaping medical imaging diagnostics, heralding a new era of accuracy and efficiency (Figure 2). AI, particularly through deep learning, plays a pivotal role in this change, enabling systems to detect abnormalities and predict disease progression with remarkable precision when trained with comprehensive data sets. The integration of MRI and PET scans illustrates the potential of multimodal imaging; by combining MRI’s anatomical detail with PET’s metabolic insights, a more complete picture of diseases is achieved, leading to better-targeted treatments. Cutting-edge real-time and high-definition imaging technologies are refining the nuances of diagnostics, offering ultra-detailed images and immediate feedback that enhance clinical decisions. Precision medicine is revolutionizing imaging diagnostics, aiming to tailor treatments to individual patients’ unique characteristics, resulting in highly personalized care plans and more effective therapies.

In summary, the future of imaging diagnostics, especially for TBM, looks promising. AI, advanced imaging techniques, and precision medicine are set to transform patient care. With ongoing innovation, we anticipate a range of new tools designed for this field, supported by interdisciplinary collaboration and global cooperation, to drive this exciting progress forward.

## 6 Conclusion

In the continually evolving landscape of modern medicine, the diagnosis and treatment of TBM remain challenging. However, recent technological advancements and breakthroughs, particularly in the field of medical imaging diagnostics, have offered us new opportunities. Through the application of cutting-edge imaging technologies and AI, we can not only diagnose more accurately but also provide more effective treatment options. The use of AI, multimodal imaging, and high-resolution techniques is rapidly transforming the realm of TBM imaging diagnosis, making it more precise and personalized. To sustain momentum in this research area, transdisciplinary collaboration and global cooperation will be key.

## Author contributions

XC: Writing – original draft, Project administration, Writing – review & editing. FC: Writing – review & editing, Project administration, Writing – original draft. CL: Writing – review & editing, Project administration, Writing – original draft. GH: Writing – review & editing, Project administration, Writing – original draft. HC: Writing – review & editing, Project administration, Writing – original draft. YW: Writing – review & editing, Data curation, Visualization. YC: Data curation, Writing – review & editing, Visualization. JS: Writing – review & editing, Data curation, Visualization. YY: Data curation, Visualization, Writing – review & editing. CD: Visualization, Writing – review & editing, Data curation. LC: Visualization, Writing – review & editing, Data curation. XW: Visualization, Writing – review & editing, Data curation. EC: Visualization, Writing – review & editing, Data curation. JW: Visualization, Writing – review & editing, Data curation. MW: Visualization, Writing – review & editing, Data curation. LZ: Visualization, Writing – review & editing, Data curation. JZ: Writing – review & editing, Data curation, Visualization. DH: Writing – review & editing, Data curation, Visualization. WP: Writing – review & editing, Data curation, Visualization. SP: Writing – original draft, Writing – review & editing, Funding acquisition, Resources. CZ: Project administration, Writing – review & editing. SQ: Funding acquisition, Resources, Writing – review & editing. FS: Funding acquisition, Resources, Writing – review & editing.

## Glossary

MRI: magnetic resonance imaging
TB: tuberculosis
TBM: tuberculous meningitis
CT: computed tomography
CTA: computed tomography angiography
MRA: magnetic resonance angiography
DSA: digital subtraction angiography
VWI: vessel wall imaging
HR-VWI: high-resolution vessel wall imaging
MRV: magnetic resonance venography
PC-MRI: phase-contrast magnetic resonance imaging
ASL: arterial spin labeling
CBF: cerebral blood flow
DTI: diffusion tensor imaging
fMRI: functional magnetic resonance imaging
MD: mean diffusivity
FA: fractional anisotropy
AI: artificial intelligence
T2WI: T2-weighted imaging
PET: positron emission tomography
